# Identification of Target Genes Involved in the Antiproliferative Effect of Enzyme-Modified Ginseng Extract in HepG2 Hepatocarcinoma Cell

**DOI:** 10.1155/2013/502568

**Published:** 2013-09-23

**Authors:** Sung-Il Jang, Yeon-Weol Lee, Chong-Kwan Cho, Hwa-Seung Yoo, Jun-Hyeog Jang

**Affiliations:** ^1^East-West Cancer Center, Dunsan Oriental Hospital of Daejeon University, Daejeon 302-122, Republic of Korea; ^2^Department of Biochemistry, Inha University School of Medicine, Incheon 400-712, Republic of Korea

## Abstract

Ginsenosides are ginseng saponins, which are the major biologically active components of *Panax ginseng*, often metabolized by intestinal bacteria into more effective forms. In this study, we found that the antiproliferative activity of ginseng increased after enzymatic processing of ginseng saponin (50% inhibitory concentration [IC_50_], >30 **μ**g/mL), which may be the result of the accumulation of minor saponins, such as Rh1, Rg3, compound K, and PPT constituents in ginseng saponin. Using the Agilent PrimeView Human Gene Expression Array, we found that the expression of several genes involved in apoptosis (*caspase-4, Annexin A2, HSPA9, AIFM1, UQCRC2*, and *caspase-7*) were increased in HepG2 human hepatocarcinoma cells after their treatment with enzyme-modified ginseng extract (EMGE). Furthermore, several genes implicated in cell cycle progression (*CDCA3, CDCA8, CABLES2, CDC25B, CNNM3*, and *CCNK*) showed decreased expression in HepG2 cells treated with EMGE. Finally, from flow cytometric analysis, we found that EMGE-treated HepG2 cells showed increased apoptotic sub-G1 population (24%), compared with that observed in DMSO-treated control cells (1.6%). Taken together, our results suggest that EMGE induces anticancer activity through the induction of apoptosis-related genes and cell cycle arrest via decreased expression of cell cycle regulatory genes.

## 1. Introduction

*Panax ginseng* (Korean ginseng) has been used as a traditional medicine for the treatment of cancer and has been shown to inhibit tumor cell proliferation and tumor growth, to induce differentiation and apoptosis, and to inhibit cancer cell invasion [1–4]. The major biologically active components of *Panax ginseng* are a series of saponin glycosides collectively known as the ginsenosides, a group of steroidal saponins. Over 50 ginsenosides have been isolated and identified from ginseng saponins to date [5]. 

Ginsenosides are characterized by a steroid-like skeleton consisting of 4 *trans*-rings, with modifications that depend on the type (e.g., glucose, maltose, and fructose) and number of sugar moieties, as well as the sites of attachment of the hydroxyl group (e.g., C-3, C-6, or C-20). Based on chemical structural characteristics, ginseng saponins can be divided into protopanaxadiol and protopanaxatriol groups, except for ginsenoside Ro, which is derived from an oleanolic group. In the protopanaxadiol group, sugars are attached to the *β*-OH at C-3 and another –OH at C-20, as found in Rb1, Rb2, Rc, Rd, Rg3, and Rh2. In the protopanaxatriol group, sugar residues are attached to the *α*-OH at C-6, with another –OH at C-20, examples being Re, Rg1, Rg2, Rh1, and Rf. 

Recent studies have indicated that intestinal bacteria cleave the oligosaccharide connected to the aglycon stepwise from the terminal sugar to form the ginsenoside metabolite 20*S*-protopanaxadiol 20-*O-*β**-d-glucopyranoside (compound K) after oral administration of ginseng extract in both humans and rats [6]. Wakabayashi et al. have revealed that the antitumor activities of ginsenoside following oral administration are attributable to the metabolites formed by colonic bacteria-mediated deglycosylation [7]. In addition, compound K is known to exhibit cytotoxicity by the induction of apoptosis and cell cycle arrest at the G_1_ phase, by a caspase-dependent pathway via mitochondrial disruption in tumor cells, and by reversing multidrug resistance in tumor cells [8–10]. The combined treatment of compound K and radiation enhances human lung cancer cell death [8, 11].

Various transformation methods, including mild acid hydrolysis, enzymatic conversion, and microbial conversion, have been tested for their ability to transform ginsenosides to enhance their physiological effectiveness. In this study, we investigated the antiproliferative effects and possible mechanism of action of EMGE that were transformed by enzyme treatment, by using the human hepatocarcinoma cell line HepG2.

## 2. Materials and Methods

### 2.1. Cell Culture

HepG2 cells were maintained in Dulbecco's Modified Eagle's Medium (DMEM) supplemented with 10% heat-inactivated fetal calf serum (Invitrogen, Carlsbad, CA), 100 units/mL penicillin G sodium, 100 *μ*g/mL streptomycin sulfate, and 0.25 *μ*g/mL amphotericin B (Invitrogen, Carlsbad, CA) in a 5% CO_2_ atmosphere at 37°C. Confluent cells were detached with 0.25% trypsin-EDTA for 5 min, and aliquots were subcultured. Human hepatoma HepG2 cells were purchased from the Korean Cell Line Bank (KCLB, Seoul, Republic of Korea). 

### 2.2. Preparation of Enzyme-Modified Ginseng Extract (EMGE)

The root of regular ginseng (4 years old) was purchased from G&V, Korea. A total of 20 g of pulverized ginseng root powder were suspended in 380 mL of distilled water and then sterilized by heating at 121°C for 15 min. To reduce the complexity of components in ginseng root, the extract was fractionated via extraction with water, methanol, and butanol. Among the fractions, ginseng butanol extract contained the compounds with specific anticancer activity. In addition, the suspension was treated with aliquots of filter-sterilized commercial enzymes (laminarinase 100 mg, pectinase 100 mg) with an equimolar ratio (1 : 1, specific activity unit). For these treatments, the mixture was incubated at 40°C for 2 days and then evaporated to dryness at 60°C. These enzyme-modified ginseng powders were then suspended in 400 mL of 80% (v/v) methanol and subjected to ultrasonication for 5 min followed by filtration through Whatman No. 2 Filter Paper. The wet powder on the filter paper was then collected, suspended, treated in an ultrasound bath, and filtered in the same manner once again, after which the two filtrates were combined and evaporated to dryness at 50°C. The methanol extract dissolved in 200 mL of distilled water was washed with 200 mL of ethyl acetate in a separation funnel and then extracted with 200 mL of butanol. Next, the butanol extract was evaporated to dryness at 50°C and then dissolved to a concentration of 10% (w/v) in 70% ethanol. 

### 2.3. HPLC Analysis of Ginsenosides

Standard ginsenosides including Rg1, Re, Rf, Rh1, Rb1, Rc, Rb2, f1, Rd, Rg3(S), Rg3(R), PPT, Com K, Rh2, and PPD were obtained from ChromaDex Inc. (Irvine, CA) and analyzed using an Acquity UPLC system (Waters, Milford, MA) with an Acquity BEH C_18_ HPLC column. The mobile phase consisted of solution A (CH_3_CN, HPLC-grade, JT Baker, NJ) and solution B (Milli-Q H_2_O, Millipore, MA). The flow rate was set at 0.6 mL/min, and the injection of volume was fixed at 2 : 1. The separation of ginsenosides was performed using an isocratic gradient of 100% solution A for 27 min. The column temperature was maintained at room temperature during the separation, and the UV diode array detection was set at 203 nm. To confirm a reliable retention time of sample ginsenoside, the individual ginsenosides were identified and quantified based on comparison with the retention times of standard ginsenosides.

### 2.4. Cell Viability Assay

The effect of EMGE on cell viability was determined using the 3-(4,5-dimethylthiazol-2-yl)-2,5-diphenyltetrazolium bromide (MTT, a yellow tetrazole) assay, which quantifies the number of viable cells. Cells were seeded (2 × 10^4^ cells/well) in 24-well flat-bottomed plates (Nunc, EU). After incubation at 37°C for 24 h, the medium was replaced with EMGE at the appropriate concentrations. Control cells were treated with DMSO at a final concentration of less than 0.2%. After further 24 h incubation, cells were washed 3 times with Dulbecco's phosphate-buffered saline (DPBS), and 500 *μ*L MTT solution (5 mg/mL in PBS) was added to each well. After incubating for 4 h, the medium was removed, and 200 *μ*L of formazan (crystals dissolved in DMSO) was added to the cells. The absorbance of each well was read at 540 nm using a microplate reader.

### 2.5. RNA Extraction and cDNA Synthesis

Total RNA was extracted using the Easy-spin RNA Extraction kit (iNtRON, Seoul, Republic of Korea) according to the manufacturer's instructions. The purity of RNA was assessed by measuring the absorption at 260 and 280 nm (values of the ratio of *A*_260_/*A*_280_ of 1.9–2.1 were considered acceptable) and by ethidium bromide staining of 18 S and 28 S RNA by gel electrophoresis. RNA concentrations were determined from the *A*_260_. Two micrograms of total RNA were reverse-transcribed in a 20 *μ*L reaction mixture containing 50 units of SuperScript II reverse transcriptase (Invitrogen, Carlsbad, CA), 5 *μ*M DTT, 40 units of RNaseOUT recombinant ribonuclease inhibitor (Invitrogen, Carlsbad, CA), 0.5 *μ*M of random hexanucleotide primers, and 500 *μ*M of dNTP mixture. The reverse transcription reaction was carried out at 50°C for 60 min. The reaction was terminated by heating the reaction mixture at 70°C for 15 min, and the cDNA was stored at −20°C.

### 2.6. Quantitative Real-Time Reverse Transcriptase Polymerase Chain Reaction (RT-PCR)

Overexpression of the target genes was confirmed by quantitative real-time RT-PCR. All real-time PCR analyses were performed on an ABI Step One Real-time PCR system (Applied Biosystems). Each reaction contained 0.1 *μ*M of each primer, 10 *μ*L of 2x SYBR Green PCR master mix (including AmpliTaq Gold DNA polymerase with buffer, dNTPs mix, SYBR Green I dye, ROX dye, and 10 mm MgCl_2,_ Applied Biosystems), and 1 *μ*L of the template cDNA in a 20 *μ*L total reaction volume. The typical amplification program included activation of the enzyme at 94°C for 10 min, followed by 40 cycles of denaturation at 94°C for 15 s, then annealing and extension at 60°C for 1 min. The *C*_*T*_ (cycle threshold) value for each gene was determined using the automated threshold analysis function of the ABI instrument and normalizing to *C*_*T*(G3PDH)  _ to obtain d*C*_*T*_ ( = *C*_*T*(G3PDH)_ − *C*_*T*(test)_). The difference of *n* between the 2*C*_*T*_ or d*C*_*T*_ values indicates a 2^n^-fold difference in the amount of the target sequence between the 2 cDNA samples being compared. The primers used in quantitative PCR are shown in Table 1.

### 2.7. Microarray Assay

Gene expression was analyzed with Agilent PrimeView Human Gene Expression Array containing 36,000 genes transcript and variants via Unigene annotation (Affymetrix, Santa Clara, CA), which are comprised of over 45,000 probe sets representing approximately 38,500 well-characterized human genes. For each gene, 11 pairs of oligonucleotide probes were synthesized *in situ* on the arrays. Biotinylated cRNA was prepared according to the standard Affymetrix protocol from 100 ng of total RNA (Expression Analysis Technical Manual, 2001, Affymetrix). Following fragmentation, 12 *μ*g of aRNA was hybridized for 16 h at 45°C on a GeneChip Human Genome Array. GeneChips were washed and stained in the Affymetrix Fluidics Station 450. GeneChips were scanned using the Affymetrix GeneChip Scanner 3000 7G. The data were analyzed with Microarray Suite version 5.0 using the Affymetrix default analysis settings and global scaling as the normalization method. The trimmed mean target intensity of each array was arbitrarily set to 100. The normalized and log transformed intensity values were then analyzed using GeneSpring GX 11.5.1 (Agilent Technologies, CA). Fold-change filters included the requirement that the genes be present in at least 200% of controls for genes with upregulated expression and less than 50% of controls for genes with downregulated expression. Hierarchical cluster analysis using GeneSpring GX 11.5.1 (Agilent technologies, CA) identified clustered groups as those that behaved similarly across experiments. The clustering algorithm was Euclidean distance, with average linkage.

### 2.8. Cell Count and Flow Cytometry Analysis

Cell counts were performed using a hemocytometer. Approximately 1 × 10^6^ HepG2 cells were suspended in 100 *μ*L of PBS, and 200 *μ*L of 95% ethanol was added while vortexing. The cells were incubated at 4°C for 1 h, washed with PBS, and resuspended in 250 *μ*L of 1.12% sodium citrate buffer (pH 8.4) together with 12.5 *μ*g of RNase. Incubation was continued at 37°C for 30 min. The cellular DNA was then stained by applying 250 *μ*L of propidium iodide (50 *μ*g/mL) for 30 min at room temperature. The stained cells were analyzed by fluorescence-activated cell sorting (FACS) flow cytometry (FACScanner, Becton Dickinson) for relative DNA content based on red fluorescence.

## 3. Results

### 3.1. Chromatographic Analysis of Ginseng Saponin during the Enzyme Treatment

Ginseng saponins (ginsenosides) have been regarded as the principal components responsible for the pharmacological activities of ginseng, and a large number of ginsenoside derivatives have been identified in *Panax ginseng *[5]. Recently, cytotoxic effects of minor saponins, such as Rh1 and Rh2, on the growth of various cancer cells and also inhibitory effects of human intestinal bacterial saponin metabolites, such as compound K and protopanaxatriol (PPT), on the growth, invasion, and migration of tumor cells, have been reported [12–16]. High-performance liquid chromatography (HPLC) analysis was performed to determine the changes in chemical constituent in ginseng saponin following enzyme treatment. HPLC results showed the appearance of minor ginsenosides (Rh1, Rg3, and compound K), indicative of EMGE (Table 2). 

### 3.2. Antiproliferative Effects of EMGE on HepG2 Hepatocarcinoma Cells

**Figure 1:**
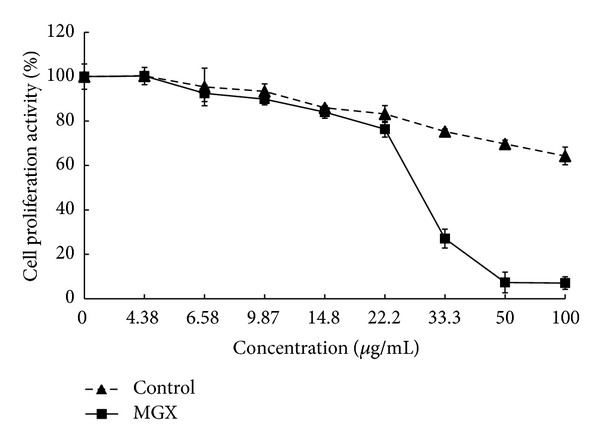
Antiproliferative effects of EMGE on human HepG2 hepatocarcinoma cells. Cell viability at the indicated concentrations of EMGE at 24 h was assessed by the MTT test. The treated cells were used for total RNA isolation followed by microarray analysis.

Ginsenosides have been shown to inhibit tumor cell proliferation and tumor growth and to induce differentiation and apoptosis [17]. To determine the antiproliferative effects of EMGE on HepG2 human hepatocarcinoma cells, we performed the MTT assay. EMGE inhibited HepG2 cell growth in a dose-dependent manner at 14.8, 22.2, 33.3, 50, and 100 *μ*g/mL at 24 h. The concentration at which 50% growth inhibition (IC_50_) occurred was 30 *μ*g/mL (Figure 1). However, exposure of HepG2 cells to 30 *μ*g/mL nontreated ginseng saponin extract showed less growth inhibition (approximately 20%). Thus, these results suggest that enzymatic processing of ginseng saponin increased its antiproliferative effects on HepG2 cells.

### 3.3. Identification of Differentially Expressed Genes in HepG2 Human Hepatocarcinoma Cells Treated with EMGE

Although ginseng has been used as a traditional medicine for the treatment of cancer, the underlying mechanisms for the varying activities observed following exposure to ginseng are largely unclear. To identify relevant alterations of gene expression associated with treatment of EMGE, we analyzed the gene expression profiles of HepG2 cells by using Affymetrix Gene Chip arrays containing more than 38,500 genes. From the results of Affymetrix Gene Chip array analysis, we detected 31 differentially expressed genes exhibiting at least a 5-fold change following treatment with EMGE. Of these, 17 had significantly upregulated expression, and 14 had significantly downregulated expression when comparing treatment with the EMGE versus the control treatment (Tables 3 and 4). 

Several genes involved in apoptosis (*caspase-4*, *Annexin A2*, *HSPA9*, *AIFM1*, *UQCRC2*, and *caspase-7*) were expressed at higher levels following treatment with EMGE (Table 3). The expression of genes involved in the regulation of cell signaling (*MAPK6*, *SMAD2*, *IL6ST*, *KLF6*, and *IFITM1*) also increased. Furthermore, several genes implicated in cell adhesion (*CTNNB1*), transport (*AQP3*, *IFITM1*), and the immune response (*IL8*) showed increased expression in HepG2 human hepatocarcinoma cells that were treated with EMGE. 

In contrast, 14 genes showed reduced expression in HepG2 human hepatocarcinoma cells treated with EMGE. There was an overrepresentation of members of genes involved in cell cycle progression (*CDCA3*, *CDCA8*, *CABLES2*, *CDC25B*, *CNNM3*, and *CCNK*) and cellular metabolism (*CS*, *ALDH3B1*, and *NPRL3*).  Table 4 lists the names of genes exhibiting a reduction in expression, where the difference was >5-fold. This group of genes that were expressed very differently may serve as an important target in EMGE-mediated anticancer activity in human hepatocarcinoma cells. 

### 3.4. Real-Time RT-PCR Validation of Microarray Data

To confirm the results obtained using microarrays, we choose to determine if the expression of 5 target genes was regulated by EMGE in HepG2 cells by quantitative real-time RT-PCR analysis. These genes included 3 genes with highly upregulated expression (*cyp450*, *caspase-4*, and *caspase-7*) and 2 with highly downregulated expression (*Cyclin K* and *Cyclin M*). To accurately quantify the expression of these 5 genes, *GAPDH* expression was amplified and the mean data in each case was used to normalize the result. 

**Figure 2:**
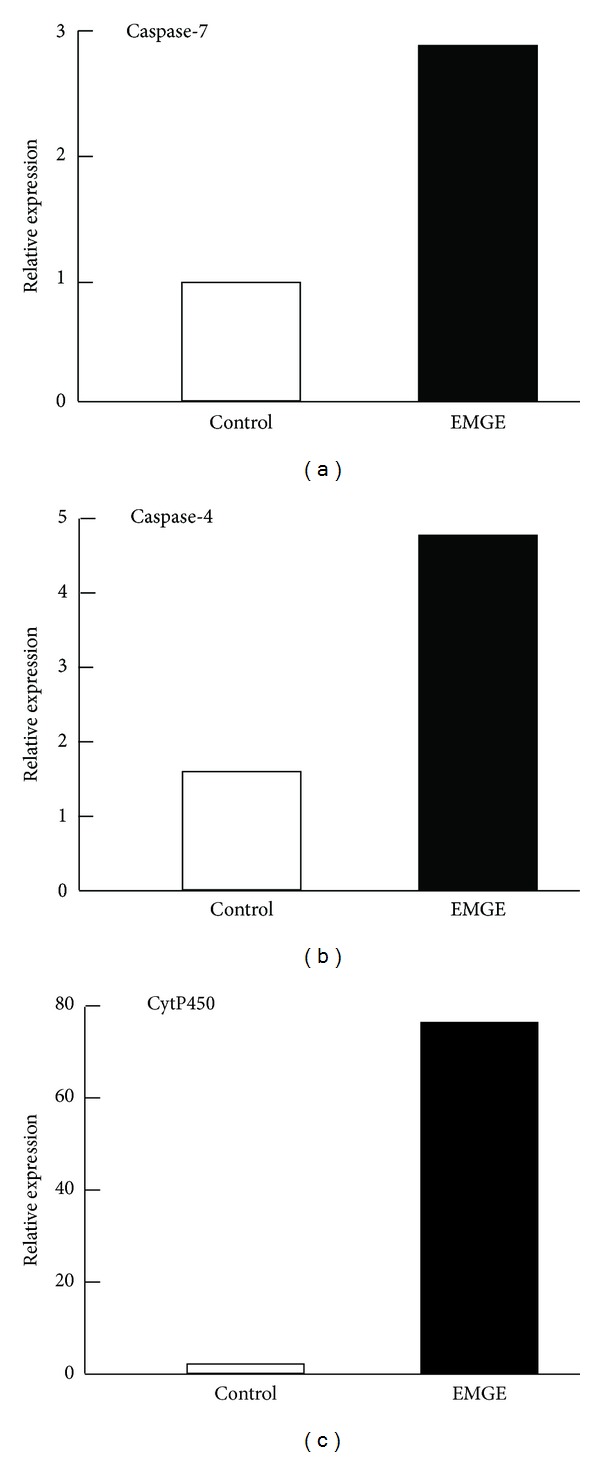
Quantitative real-time RT-PCR analysis of 3 genes with upregulated expression: *cyp450*, *caspase-4*, and *caspase-7*.

**Figure 3:**
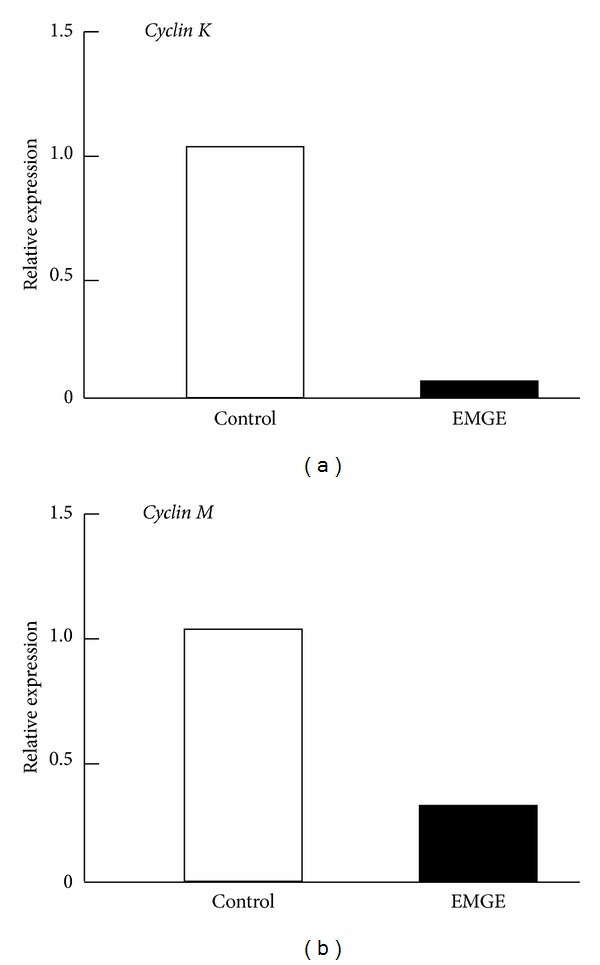
Quantitative real-time RT-PCR analysis of 2 downregulated genes: *Cyclin K* and *Cyclin M*.

As shown in Figures 2 and 3, the expression level of the 3 selected upregulated genes (*cyp450* (73-fold), *caspase-4* (6-fold), and *caspase-7* (3-fold)) significantly increased upon treatment with EMGE. Conversely, the expression of the 2 downregulated genes (*Cyclin M* (4-fold), *Cyclin K* (12-fold)) decreased. These results indicate that the real-time RT-PCR results are highly consistent with the microarray data.

### 3.5. Effect of EMGE on Cell Cycle Distribution and Apoptosis in HepG2 Hepatocarcinoma Cells

**Figure 4:**
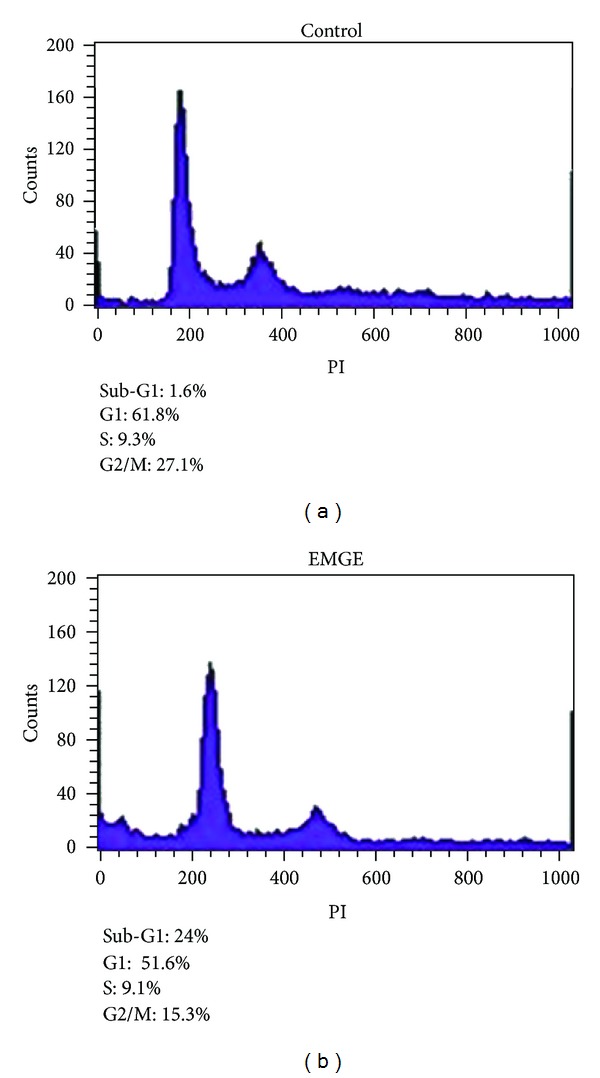
FACS analysis of PI-stained nuclei of HepG2 cells treated with EMGE. Cells were treated with DMSO (0.05%) or EMGE (50 *μ*g/mL) for 24 h, and cell cycle analysis was conducted as described in Section 2. Data are representative of 3 independent experiments.

To determine whether EMGE has an effect on cell cycle arrest, apoptosis, or both in human HepG2 hepatocarcinoma cells, cells treated with DMSO or EMGE for 12 or 24 h were subjected to flow cytometric analysis after DNA staining. Histograms of flow cytometric data are shown in Figure 4. Control cells (DMSO-treated) progressed through the cell cycle normally. In contrast, EMGE-treated HepG2 cells showed increased apoptotic sub-G1 populations (24%) compared with that observed in DMSO-treated control cells (1.6%). These results suggest that EMGE induces antiproliferative effects in HepG2 cells, mainly through apoptotic mechanisms. 

## 4. Discussion

We found that the enzymatic processing of ginseng saponin could increase the content of active constituents and enhance its anticancer activity, presumably because of the production of minor saponins, such as Rh1, Rg3, compound K, and PPT constituents in ginseng saponin. In this study, we used cDNA microarray analysis to detect target genes involved in the anticancer activity of EMGE. Microarray (DNA chip) is a 2D array of microscopic DNA spots for high-throughput expression screening. We found that several genes involved in apoptosis (*caspase-4*, *Annexin A2*, *HSPA9*, *AIFM1*, *UQCRC2*, and *caspase-7*) as well as several genes involved in cell signaling (*MAPK6*, *SMAD2*, *IL6ST*, *KLF6*, and *IFITM1*) were expressed at higher levels after treatment with EMGE. Furthermore, several genes implicated in cell cycle progression (*CDCA3*, *CDCA8*, *CABLES2*, *CDC25B*, *CNNM3*, and *CCNK*) and metabolism (*CS*, *ALDH3B1*, and *NPRL3*) exhibited decreased expression in HepG2 human hepatocarcinoma cells treated with EMGE. Thus, the apoptosis-related genes with upregulated expression and cell cycle-related genes with downregulated expression could be important target genes involved in the anticancer activity of EMGE. 

In this study, *caspase-4* and *caspase-7*, which are components of the cell-intrinsic apoptosis machinery, were significantly induced by EMGE. *Caspase-4* has a functional role in endoplasmic-reticulum-stress- (ERS-) induced apoptosis in humans [18]. Upregulation of *caspase-4* has been demonstrated to mediate apoptosis in human bronchial epithelial cells [19], human neuroblastoma SKN-SH cells, and human esophageal squamous carcinoma EC109 [20]. Thus, the induction of apoptosis-related genes, such as *caspase-4* and *caspase-7*, in cells treated with EMGE may contribute to the antiproliferative activity of EMGE. 

The cytochrome P450 1A1 (CYP1A1) is a member of the cytochrome P450 monooxygenase superfamily, which plays an important role in xenobiotic metabolism as well as in carcinogenesis [21]. In this study, CYP1A1 level was significantly increased after treatment with EMGE. The CYP1A1 enzymes mediate the bioactivation of carcinogens found both in the human diet and in the environment, including compounds such as polycyclic aromatic hydrocarbons, such as 3-methylcholanthrene, benzo(a)pyrene, polyhalogenated aromatic hydrocarbons, and certain congeners of polyhalogenated biphenyls [22]. Our finding that the EMGE increased the expression of CYP1A1 is consistent with the previous results showing the induction of CYP1A1 expression by ginsenosides Rg1 and Rb1 [23]. 

Cell cycle progression is a tightly regulated process requiring regulatory factors such as CDCs and cyclins. Thus, lack of cell cycle machinery molecules often causes cell cycle arrest leading to cell growth inhibition, apoptosis, or both. Our results showing the decreased expression of several genes implicated in cell cycle regulation (*CDCA3*, *CDCA8*, *CABLES2*, *CDC25B*, *CNNM3*, and *CCNK*) in HepG2 cells treated with EMGE suggest that the induction of cell cycle arrest by EMGE may contribute to the antiproliferative activity of EMGE.

In summary, we found that the anticancer activity of ginseng was increased by enzymatic processing of ginseng saponin, which may be the result of the relative accumulation of minor saponins, such as Rh1, Rg3, compound K, and PPT constituents in ginseng saponin. Moreover, the results of microarray analysis suggest that EMGE induces anticancer activity through the induction of apoptosis-related genes and the decreased expression of cell cycle regulatory genes. However, these results do not exclude the role of other genes that may be involved in anticancer activity.

## Figures and Tables

**Table 1:** Primer sequences for real-time RT-PCR experiments.

Genes	Sense (5′-3′)	Antisense (5′-3′)
*Caspase-4 *	GAAGGACAAACCCAAGGTCA	AAGAGGCCACTTCCAAGGAT
*Cyp450 *	TGATAAGCACGTTGCAGGAG	CAAAGCAAATGGCACAGATG
*Caspase-7 *	AGACCTCATCCGGTCAAGTG	TTATCAGACAGCGCAACAGC
*Cyclin M *	GAAGCGGAAGGAGGAGTTCT	CGGGCTGAATACATCCACTT
*Cyclin K *	GACATCTGCCACCAAATCCT	GCACTTGTGGTGTAGGCTGA
*GAPDH *	TGGAAGGACTCATGACCACA	TTCAGCTCAGGGATGACCTT

**Table 2:** HPLC analysis of EMGE.

	(mg/g)											
	Rg1	Re	Rf	Rh1	Rb1	Rc	Rb2	F1	Rd	Rg3	Com.K	Rh2
Control (ginseng)	9.2	7.2	2.5	—	7.7	4.7	2.3	2.0	0.6	—	—	—
EMGE	0.6	1.0	—	2.7	2.7	—	—	—	—	9.5	4.7	2.7

**Table 3:** Genes with highly upregulated expression due to EMGE in HepG2.

Symbol	Gene description	Fold change	GenBank ID
*CYP1A1 *	Cytochrome P450, family 1, subfamily A	20.0	NM000499
*HGD *	Homogentisate 1,2-dioxygenase	15.3	NM000187
*AQP3 *	Aquaporin 3	14.5	NM004925
*IL8 *	Interleukin 8	14.3	NM000584
*ANXA2 *	Annexin A2	11.1	NM001002857
*HSPA9 *	Heat shock 70 kDa protein 9	10.5	NM004134
*CASP4 *	Caspase-4	10.4	NM001225
*SMAD2 *	SMAD family member 2	9.8	NM001003652
*MAPK6 *	Mitogen-activated protein kinase 6	9.3	NM002748
*KLF6 *	Kruppel-like factor 6	9.0	NM001160124
*CTNNB1 *	Catenin (cadherin-associated protein), beta 1, 88 kda	8.3	NM001098209
*AIFM1 *	Apoptosis-inducing factor, mitochondrion-associated, 1	7.8	NM001130846
*PPHLN1 *	Periphilin 1	7.5	NM001143787
*UQCRC2 *	Ubiquinol-cytochrome c reductase core protein II	7.4	NM003366
*IL6ST *	Interleukin 6 signal transducer	6.4	NM001190981
*IFITM1 *	Interferon-induced transmembrane protein 1	5.1	NM003641
*CASP7 *	Caspase-7	5.0	NM001227

**Table 4:** Genes with highly downregulated expression due to EMGE in HepG2.

Symbol	Gene description	Fold change	GenBank ID
*CDCA3 *	Cell division cycle associated 3	−8.4	NM031299
*CS *	Citrate synthase	−8.0	NM004077
*CDCA8 *	Cell division cycle associated 8	−7.7	NM018101
*CABLES2 *	Cdk5 and Abl enzyme substrate 2	−7.4	NM031215
*CDC25B *	Cell division cycle 25 homolog B	−6.6	NM004358
*CNNM3 *	Cyclin M3	−6.2	NM017623
*ZNF362 *	Zinc finger protein 362	−5.8	NM152493
*CCNK *	Cyclin K	−5.8	NM001099402
*RAD51AP1 *	RAD51 associated protein 1	−5.8	NM001130862
*LEAP2 *	Liver expressed antimicrobial peptide 2	−5.3	NM052971
*HEPACAM *	Hepatocyte cell adhesion molecule	−5.1	NM152722
*ALDH3B1 *	Aldehyde dehydrogenase 3 family, member B1	−5.0	NM000694
*NPRL3 *	Nitrogen permease regulator-like 3	−5.0	NM001039476
*DOT1L *	DOT1-like, histone H3 methyltransferase	−5.0	NM032482
